# Genomic Analysis of Retinal Development in the Mouse

**DOI:** 10.1371/journal.pbio.0020265

**Published:** 2004-06-29

The eyes may be the window to the soul for poets, but for neuroscientists, they serve a more practical purpose. Of the 100 trillion or so cells that make up the human body, over 100 billion are dedicated to the structure and operation of the brain alone. Given the molecular and functional complexity inherent in such numbers, neuroscientists have historically focused on a more tractable system, the vertebrate retina, to study central nervous system development and physiology. Cells in the retina are packaged into highly ordered anatomical layers, based on their specialized functions. This organizational structure is characteristic of other regions of the central nervous system, and allows the brain to take in and integrate sensory information simultaneously, using discrete computational units. Creating such functional microprocessors depends on making the right cell at the right place and time.

During development, cells undergo periods of proliferation and increasing specialization (differentiation), generating seven types of retinal cells (six types of neurons and one glial cell type) in a precise order at specific times. Mature, specialized cells arise from a pool of proliferating progenitors—cells that have already committed to becoming a retinal cell but haven't yet settled on a particular cell type. But progenitors are not all alike; they display intrinsic differences in their “competence” to produce a particular subset of retinal cells at a particular stage of development. These differences may help ensure that ganglion cells, for example, are established before photoreceptors, since photoreceptors rely on ganglion cells to transmit their signals to the brain.[Fig pbio-0020265-g001]

**Figure pbio-0020265-g001:**
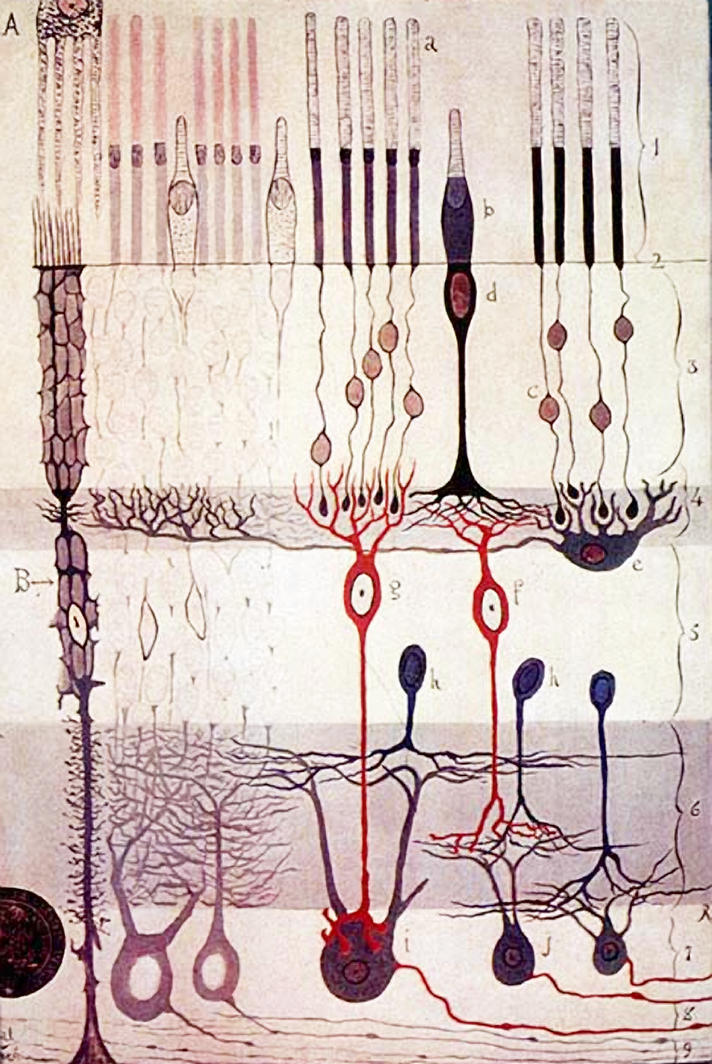
Classic drawing of the retina by Ramón y Cajal

Which path a cell ultimately chooses stems from a combination of both intrinsic competence factors—likely determined by a cell's gene expression program—and external signals from the cell's environment. Progenitors give rise to “postmitotic” cells (cells that have exited the cell cycle and ceased proliferating), which go on to express characteristics associated with a specific cell type.

Beyond this framework, the molecular underpinnings of retinal development remain obscure. Differentiated cells exhibit a gene expression program unique to their cell type, but it's not clear what accounts for underlying differences among progenitors, for example, or what factors usher retinal cells into their respective specialties. To map the genetic landscape of retinal development, Constance Cepko and colleagues looked for genes expressed in retinal cells passing through various competence levels and making cell fate choices. They determined gene expression profiles by collecting bits of gene transcripts from the retinal tissue of developing mice at two-day intervals, starting with mice entering neurogenesis and ending with mice about six and a half days old. They also collected gene expression data from postnatal day 10 and from adult mice.

The authors then examined the cellular expression patterns of 1,051 of the genes that showed dynamic patterns by genomic expression profiling. Cepko and colleagues then pegged these genes to specific cell types to create a “molecular atlas of gene expression in the developing retina.” (Though the retina has many millions of cells, different cell types can be easily identified based on their telltale shape and position in the retina.) Nearly every gene known to direct retinal cell differentiation was detected in this analysis and showed high levels of expression. Genes required for cell fate choices showed peak expression near or after cells exited the cell cycle, supporting the idea that similar controls operate to put the brakes on cell proliferation and to determine cell fate. Many uncharacterized genes were expressed only in certain progenitor subsets, making them good candidates as cell fate determinants for different subtypes of retinal cells. A promising list of candidate genes for retinal development and function appear in this molecular atlas, along with candidates for retinal disease. Since many degenerative retinal diseases stem from defects in development, these genes will help researchers focus their search for therapies. And if the eye truly is the window of the nervous system, these findings may suggest general principles of cell fate determination for the developing brain, spinal cord, and other regions of the vertebrate nervous system.

